# Role of hydrops MRI in differentiating between Menière’s disease and vestibular migraine: a prospective study

**DOI:** 10.3389/fneur.2025.1582754

**Published:** 2025-05-14

**Authors:** Anja Bernaerts, Morgana Sluydts, Vincent Liégeois, Catherine Blaivie, Floris L. Wuyts, Joost van Dinther, Andrzej Zarowski, Filip Deckers, Bert De Foer

**Affiliations:** ^1^Department of Radiology, ZAS Hospitals, Antwerp, Belgium; ^2^European Institute for ORL-HNS, Antwerp, Belgium; ^3^Lab for Equilibrium Investigations and Aerospace, University of Antwerp, Antwerp, Belgium

**Keywords:** magnetic resonance imaging, Menière’s disease, vestibular migraine, endolymphatic hydrops, diagnosis

## Abstract

**Objective:**

We investigated the effectiveness of delayed post-gadolinium (Gd) three-dimensional (3D) sampling perfection with application-optimized contrasts using different flip angle evolution (SPACE) fluid-attenuated inversion recovery (FLAIR) sequence to differentiate between Menière’s disease (MD) and vestibular migraine (VM) in a prospective study.

**Methods:**

A total of 31 patients—15 with MD (10 with definite MD and 5 with probable MD) and 16 with VM (9 with definite VM, 7 with probable VM)—were prospectively enrolled between January 2019 and December 2022. The female-to-male ratio in the MD group was 7:8, while in the VM group, it was 14:2. All patients underwent a 3D SPACE FLAIR sequence and a 3D SPACE T2 sequence 4 h after intravenous (IV) injection of a single dose of gadobutrol (1.0 mmoL/mL). Cochlear endolymphatic hydrops (CEH), vestibular endolymphatic hydrops (VEH), and asymmetrical perilymphatic enhancement (PLE) were assessed.

**Results:**

None of the VM patients showed signs of CEH, VEH, or increased PLE. However, in the MD group, only two patients had normal CEH, one patient had normal VEH, and six patients demonstrated equal PLE in both ears. The logistic regression analysis using both VEH and CEH correctly predicted all cases of MD and VM, achieving 100% diagnostic accuracy for both conditions. However, using only CEH, VEH, or PLE as diagnostic criteria resulted in misclassifications: two patients were incorrectly classified as having VM based on CEH, one based on VEH, and six based on PLE. These results highlight the superior diagnostic power of the combination of CEH and VEH in logistic regression analysis.

**Conclusion:**

The combination of CEH and VEH allows for 100% accurate identification of VM and MD. This approach facilitates a reliable differential diagnosis of MD and VM when used in the appropriate clinical setting.

**Clinical relevance statement:**

This study demonstrates that hydrops magnetic resonance imaging (MRI) can accurately differentiate MD from VM. Therefore, hydrops MRI can obviate the need for trial medication in cases with clinically ambiguous findings.

## 1 Introduction

Menière’s disease (MD) is a common inner ear disorder characterized by a clinical triad of symptoms: fluctuating low-frequency sensorineural hearing loss (SNHL), tinnitus or aural fullness, and vertigo attacks lasting at least 20 min (1). In 2015, the Bárány Society established clinical diagnostic criteria for MD, classifying the condition into two categories: definite MD and probable MD (2). The clinical diagnosis of MD is typically supported by a combination of audiological, vestibular, and electrophysiological tests. However, the lack of a definite gold standard diagnostic test complicates the diagnostic process (1).

In the past decade, it has become feasible to visualize endolymphatic hydrops (EH)—a key pathophysiological feature of MD—using delayed post-gadolinium (Gd) magnetic resonance imaging (MRI) (3, 4). Intravenous (IV) Gd administration has become the method of choice for hydrops imaging, surpassing the intratympanic (IT) method. This is typically performed using a three-dimensional (3D) sampling perfection with application-optimized contrasts using different flip angle evolution (SPACE) fluid-attenuated inversion recovery (FLAIR) sequence or a three-dimensional inversion-recovery sequence with a real reconstruction (3D-real IR) sequence (5, 6). A 3-stage cochlear endolymphatic hydrops (CEH) grading system and a 4-stage vestibular endolymphatic hydrops (VEH) grading system, in combination with increased perilymphatic enhancement (PLE), have been reported to offer the highest sensitivity and specificity for the diagnosis of MD (7, 8).

Vestibular migraine (VM) is characterized by recurrent episodes of vertigo or dizziness accompanied by headache. In most cases, the clinical features are typical and allow for a reliable diagnosis according to the criteria formulated by the Bárány Society and the International Headache Society (9). However, the diagnosis of VM can be difficult at times since vertigo attacks are associated with typical migraine symptoms in only 65% of cases. In addition, cochlear symptoms—typical of MD—can also be present in VM, leading to significant overlap between the clinical signs of VM and MD (9–11). Since VM was only recently classified as a separate headache disorder, as an appendix in 2018, many VM patients have been, and continue to be, misdiagnosed with MD. Consequently, these patients do not receive the appropriate treatment, leading to a burden on both the patients and society.

To date, only a few radiological studies have explored the use of MRI in differentiating MD from VM (10–14), with all but one being retrospective in nature. Therefore, the purpose of this study was to determine if delayed post-Gd MRI, using the combination of CEH, VEH, and increased PLE, could effectively differentiate VM patients from MD patients in a prospective study design.

## 2 Materials and methods

### 2.1 Patients

This prospective, monocentric case–control study included a total of 31 patients. The MD group comprised 15 patients, who were subdivided into 10 with definite MD and 5 with probable MD, according to the 2015 updated Bárány Society criteria (2). The VM group consisted of 16 patients, with 9 having definite VM and 7 having probable VM, according to the updated criteria described by Lempert et al. (9). The average age of the patients with MD was 56 years (s = 15 years), while the average age of the patients with VM was 46 years (s = 13 years). The female-to-male ratio in the MD group was 7:8, while in the VM group, it was 14:2.

Patient selection and classification were performed by a senior neurotologist (CB), head of the vestibular clinic, with 20 years of experience. Potential overlap cases were not incorporated in the study. Only clear cases of MD and VM were selected. The study inclusion period was from January 2019 to December 2022.

The study was conducted in accordance with the Declaration of Helsinki, and the protocol was approved by the institutional review board of our hospital (GZA study number: 191001ACADEM). All patients provided written informed consent before enrollment.

### 2.2 Clinical evaluation

A full audiovestibular diagnostic work-up was performed, including pure tone audiometry (PTA), water caloric irrigation test, sinusoidal harmonic acceleration test, video head impulse test (vHIT), and cervical and ocular vestibular evoked myogenic potentials.

### 2.3 MRI hydrops protocol

All patients underwent MRI using a 3-Tesla Magnetom SkyraFit (Siemens Healthineers, Erlangen, Germany) with a 20-channel head and neck coil, 4 h after intravenous injection of a single dose of Gd (Gadovist; Bayer-Schering Pharma, Berlin, Germany; 1.0 mmoL/mL at a dose of 0.1 mmol/kg). The imaging protocol consisted of a 3D SPACE FLAIR sequence with the following parameters: echo time (TE) = 551 ms, repetition time (TR) = 10,000 ms, inversion time (TI) = 2,600 ms, field of view (FOV) = 160 mm × 160 mm, and slice thickness = 0.8 mm. The acquisition time was 7 min 10 s. A 3D SPACE T2 sequence was also acquired for anatomical reference of the labyrinthine fluid space, with the following parameters: TE = 334 s, TR = 1,090 ms, FOV = 150 mm × 150 mm, slice thickness = 0.5 mm, and acquisition time = 5 min 22 s. Detailed imaging parameters are listed in Table 1.

**Table 1:** Imaging parameters of 3D SPACE FLAIR and 3D SPACE T2 sequences.

	3D SPACE FLAIR	3D SPACE T2
TR (ms)	10,000	1,090
TE (ms)	551	334
TI (ms)	2,600	NA
FOV (mm^2^)	160 × 160	150 × 150
Matrix	250 × 256	320 × 320
Number of slices	36	112
Acquired slice thickness (mm)	0.8	0.5
Averages	2	2
Parallel imaging	Grappa factor 2	Compressed sensing factor 4.5
RF bandwidth (Hz/pixel)	501	289
Turbo factor	235	90
Echo train duration (ms)	1,113	644
Flip angle mode	Constant 120°	Constant 110°
Acquisition time	7 min 10 s	5 min 22 s

### 2.4 Imaging analysis

The MR images were analyzed prospectively in consensus by two experienced head and neck radiologists (AB and BDF), each with 20 and 30 years of experience in head and neck radiology, respectively. The radiologists were blinded to all scan parameters, clinical findings, and whether the symptoms were unilateral (affecting one side) or bilateral (affecting both sides).

The assessment was performed qualitatively and included the evaluation and grading of CEH, VEH, and visual comparison of PLE. We used the modified EH grading system, as outlined by Bernaerts et al., for CEH (no hydrops, grade I, grade II) and VEH (no hydrops, grade I, grade II, or grade III) (7). PLE was classified as being less than, equal to, or more than, as described in the 2019 Bernaerts study (7).

### 2.5 Statistical analysis

Using SPSSv29, we performed a logistic regression analysis that included the following variables: age (in years), CEH, VEH, PLE, and gender. The aim was to identify which parameters were significant in correctly predicting the diagnosis of MD and VM. The association between specific variables and the occurrence of VM or MD was investigated using the chi-squared test, while Student’s *t*-test was applied to compare continuous variables between the two cohorts. The Mann–Whitney U test was used when the data were not normally distributed.

## 3 Results

**Figure 1:**
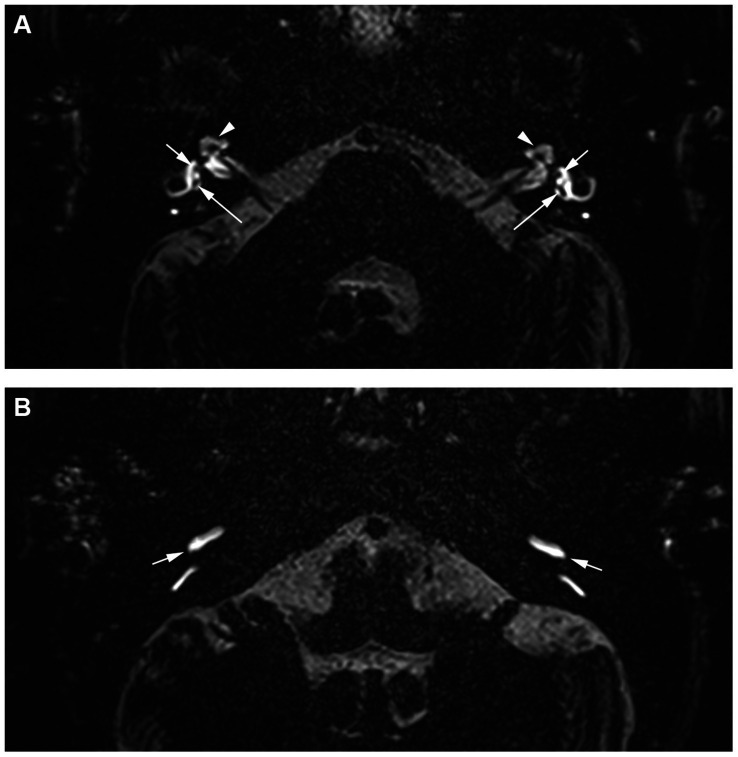
A 66-year-old woman with a history of almost daily short attacks of vertigo lasting up to 10 min, accompanied by nausea. These attacks are followed by pronounced headaches lasting for the rest of the day, accompanied by photophobia and phonophobia. During these attacks, she also experiences pressure in both ears with bilateral tinnitus but no hypoacusis. **(A)** An axial 4 h-delayed Gd-enhanced 3D SPACE FLAIR sequence at the level of the vestibule and the apical turn of the cochlea. There is a normal slit-like appearance of the scala media in the apical turn of the cochlea on both sides (arrowhead). A normal, small, and separately visible saccule is located anterior and medial in the vestibule on both sides (small arrow), and a larger utricle is positioned more posterior and lateral (large arrows). **(B)** An axial 4 h-delayed Gd-enhanced 3D SPACE FLAIR sequence of the basal turn of the cochlea and the posterior semicircular canal shows symmetrical PLE in the basal turn of the cochlea. In conclusion, the absence of CEH, VEH, and asymmetrical PLE supports the diagnosis of VM.

**Figure 2:**
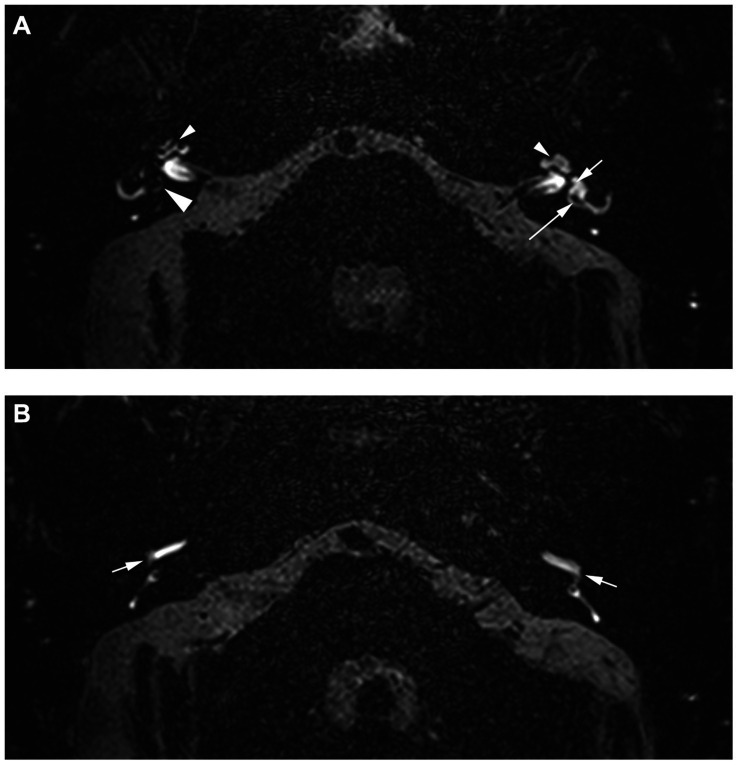
A 60-year-old man experiencing episodic attacks of vertigo with nausea and vomiting for the past 5 years. In addition, he also experiences tinnitus, a pressure sensation, and hearing loss on the right sight. Headaches and/or migraines are not reported. **(A)** An axial 4 h-delayed Gd-enhanced 3D SPACE FLAIR sequence at the level of the vestibule and the apical turn of the cochlea. There is a band-like area of signal loss in the apical turn of the cochlea on the right (small arrowhead), corresponding to complete obliteration of the scala vestibuli by the hydropic cochlear duct, consistent with a CEH grade II. This is in contrast to the normal slit-like appearance of the scala media on the left (small arrowhead). The vestibule on the right side is entirely filled up with the enlarged and fused saccule and utricle (large arrowhead), consistent with a VEH grade III. In contrast, the normal left side shows a separately visible saccule located anteriorly and medially (small arrow) and a posteriorly located utricle (large arrow). **(B)** An axial 4 h-delayed Gd-enhanced 3D SPACE FLAIR sequence at the level of the basal turn of the cochlea and the posterior semicircular canal. Increased PLE is observed in the basal turn of the cochlea on the right side, compared to the left (arrows). In conclusion, the increased PLE on the right, in combination with the grade II CEH and grade III VEH, is nearly pathognomonic for the diagnosis of (definite) MD.

None of the participants classified as having probable or definite VM (0/15) showed any signs of CEH, VEH, or increased PLE (Figure 1). In the MD group, two patients had no CEH, one patient had no VEH, and six patients had equal PLE. However, all MD patients had at least one abnormality (Figure 2). Asymmetrical PLE was observed more frequently in probable cases than in definite MD cases (Table 2). No cases of endolymphatic hydrops were found in the contralateral, asymptomatic ear of the MD patients, except in one case.

**Table 2:** MRI characteristics of 31 patients with VM (16) and MD (15), including CEH, VEH, and PLE.

	Grade	Definite MD	Probable MD	Definite VM	Probable VM
CEH	Normal	1	1	9	7
	CEH: grade I	4	2	0	0
	CEH: grade II	5	2	0	0
VEH	Normal	1	0	9	7
	VEH: grade I	1	2	0	0
	VEH: grade II	5	3	0	0
	VEH: grade III	3	0	0	0
PLE	Equal	5	1	9	7
	Increased PLE	5	4	0	0

Logistic regression analysis revealed that the combination of CEH and VEH resulted in 100% correct classification of MD and VM patients. Including PLE in the logistic regression analysis did not improve diagnostic accuracy.

In the MD group, the time interval since the last attack was 24 days (se = 11 days) for the group with equal PLE and 23 days (se = 8 days) for the group with increased PLE, which was not significantly different. Similarly, the duration of the disorder was not significantly associated with PLE.

## 4 Discussion

Vestibular migraine is a disorder characterized by episodes of vertigo and typical migraine symptoms. It is the most frequent type of episodic vertigo. In most cases, the clinical features are typical and allow for a reliable diagnosis based on the consensus criteria of the Bárány Society and the International Headache Society (2, 9). However, diagnosing VM can be challenging due to a considerable overlap with MD, particularly when the diagnosis is based on clinical presentation and audiovestibular function tests alone (10, 11). Patients with VM experience short and/or long episodes of severe vertigo, often without accompanying headache. They have heightened sensitivity to sound and light, and their vestibular symptoms tend to worsen during motion. VM attacks typically occur spontaneously.

Over the past decade and a half, hydrops MRI has gained increasing importance in the diagnosis of EH in patients with MD. IV Gd administration has been widely adopted as the method of choice for hydrops imaging, surpassing the IT method. This is typically performed using a heavily T2-weighted FLAIR sequence—most commonly a 3D SPACE FLAIR (5)—or a 3D-real inversion recovery (IR) sequence (6). The combination of a 3-stage CEH grading system, a 4-stage VEH grading system, and PLE evaluation has been shown to provide the highest sensitivity and specificity for the diagnosis of MD (7, 8).

Numerous studies attempting to differentiate between VM and MD using hydrops MRI are retrospective. Moreover, a large and heterogeneous group of non-MD patients (12, 13)—such as those with vertigo-associated inner ear disorders (12) or intracanalicular schwannomas (13)—is often included, making conclusions challenging, unreliable, and frequently statistically not significant.

The results of our study demonstrated that VM patients did not exhibit EH or increased PLE, enabling the differentiation between MD and VM. This is in contradiction to a previous study by Kirsch et al. (14) which reported the presence of EH in VM patients. In the study by Kirsch et al., EH in their VM group was more frequently observed in the vestibule, often bilaterally, and graded at a lower level. PLE was not evaluated in their study. This difference could be explained by the fact that in our study, patient selection was conducted by a single experienced neurotologist, who excluded patients with overlapping symptomatology, resulting in groups of clear MD and clear VM cases. In our study, clinical nystagmus evaluation was performed using video-oculoscopy, whereas Kirsch et al. used Frenzel glasses. This difference in methodology may have contributed to a clearer patient selection in our study. In the study by Gürkov et al., this might be explained by the fact that four patients with VM and EH also met the diagnostic criteria for MD—three for definite MD and one for probable MD (11).

In our study, asymmetrical PLE was only found in MD patients and was relatively more frequent in probable MD cases than in definite MD cases. In previous studies, PLE has been shown to be a reliable biomarker for MD (7). It is also considered a good indicator for assessing MD activity (15). We did not find a correlation between the time since the last attack and PLE, possibly due to the equally short time interval between the last attack and MRI in both subgroups. This represents a logistical advantage of the study site.

None of the VM patients showed signs of CEH, VEH, or increased PLE. Given the already established high sensitivity (84.6%) and specificity (92.3%) of the combination of EH and PLE in the diagnosis of MD (7, 8), the absence of these findings in VM patients enhances MRI’s effectiveness in making a diagnosis and differentiating VM from MD, with a 100% correct classification rate for both MD and VM, using the combination of CEH and VEH. In this study, PLE did not have an added value.

To diagnose VM, clinicians have to follow the diagnostic criteria established by the Bárány Society and the International Headache Society, and these criteria do not include, at this stage, objective biomarkers. Since both VM and MD are characterized by sudden attacks of vertigo, it is understandable that misdiagnosis can occur, with VM patients treated as having MD or vice versa, although the latter may be less common. Typically, when both VM and MD are plausible clinical diagnoses, a prophylaxis drug treatment protocol may be considered. If successful, the response can help distinguish between VM and MD, depending on the medication used. However, this process can be lengthy and has only a 50% chance of success. In contrast, MRI hydrops can resolve this ambiguity instantly, without the need for a trial medication.

One of the major limitations of our study is its limited number of participants. The prospective nature of the study along with strict application of the selection criteria for VM and MD, combined with disruptions caused by the pandemic, contributed to the limited sample size. Nevertheless, even with the limited sample size, the results seem to be straightforward. Further prospective clinical studies with larger patient cohorts and strict adherence to clinical inclusion criteria are required to confirm and validate these findings.

We conclude that, since CEH, VEH, and asymmetrical PLE are not found in VM patients, hydrops MRI can effectively facilitate the differential diagnosis between VM and MD when applied in the appropriate clinical setting.

## Data Availability

The original contributions presented in the study are included in the article/supplementary material, further inquiries can be directed to the corresponding author.
